# Combined procedure with radial probe and convex probe endobronchial ultrasound

**DOI:** 10.1111/1759-7714.14622

**Published:** 2022-08-29

**Authors:** Hyun Sung Chung, Kyoungjune Pak, Geewon Lee, Jung Seop Eom

**Affiliations:** ^1^ National Cancer Center, Division of Pulmonology Center for Lung Cancer Goyang Republic of Korea; ^2^ Department of Nuclear Medicine Pusan National University School of Medicine Busan Republic of Korea; ^3^ Department of Radiology Pusan National University School of Medicine Busan Republic of Korea; ^4^ Department of Internal Medicine Pusan National University School of Medicine Busan Republic of Korea; ^5^ Biomedical Research Institute Pusan National University Hospital Busan Republic of Korea

**Keywords:** bronchoscopy, diagnosis, lung neoplasms, lymph nodes, ultrasound

## Abstract

**Background:**

Concurrent bronchoscopy using radial probe and convex endobronchial ultrasound (RP‐ and CP‐EBUS) is used to simultaneously evaluate both peripheral lung lesions for the histological diagnosis of the primary tumor and mediastinal lymph nodes for mediastinal staging. So far, little is known about the combined procedure with RP‐ and CP‐EBUS.

**Methods:**

Between January 2020 and March 2021, the bronchoscopy database was reviewed to identify the clinical outcomes of the combined procedure with RP‐ and CP‐EBUS. Patients who underwent transbronchial biopsy using RP‐EBUS alone were classified as the RP‐EBUS group, while those who underwent a combined procedure with RP‐ and CP‐EBUS were classified as the combination group.

**Results:**

The overall diagnostic yield of the bronchoscopic procedure in the combination group was significantly higher than the RP‐EBUS group (90.7% vs. 70.0%, *p* < 0.001). CP‐EBUS increased the diagnostic yield of the bronchoscopic procedure in the combination group by 9.3%. Although the mean procedure time was significantly longer, and the mean doses of midazolam and fentanyl were significantly higher in the combination group (*p* < 0.001), there were no differences in the overall complication rates between the two study groups (1.4% and 1.0% for the RP‐EBUS and combination groups, respectively, *p* = 0.766).

**Conclusions:**

Combined bronchoscopy using RP‐ and CP‐EBUS is feasible and safe. In addition to mediastinal staging, CP‐EBUS increased the overall diagnostic yield of the bronchoscopic procedure by 9.3%.

## Graphical abstract

The combined procedure with radial probe TBLB and convex probe endobronchial ultrasound is feasible and safe. The combined procedure increased the overall diagnostic yield of bronchoscopy, besides concurrent diagnosis and mediastinal staging.

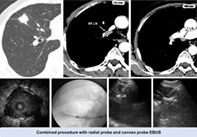

## INTRODUCTION

Endobronchial ultrasound (EBUS) techniques expand the view of bronchoscopy beyond the central airway using convex probe EBUS (CP‐EBUS), and allow the investigation of peripheral lung lesions (PLLs), where conventional bronchoscopy is not possible, using radial probe EBUS (RP‐EBUS).^1^, ^2^, ^3^, ^4^ Although mediastinoscopy is considered the gold standard for mediastinal lymph node (LN) sampling, transbronchial needle aspiration using CP‐EBUS (CP‐EBUS‐TBNA) is now being used more commonly because of its minimal invasiveness and 86%–94% accuracy.^1^ In addition, percutaneous needle aspiration or video‐assisted thoracoscopic surgery have traditionally been used for the histological diagnosis of small peripheral lung cancers, but recent developments in bronchoscopy, including transbronchial biopsy using RP‐EBUS (RP‐EBUS‐TBB) and virtual bronchoscopy navigation, have enabled the diagnosis of early‐stage lung cancers with low complication rates.^2^, ^3^, ^4^, ^5^

The introduction of low‐dose computed tomography (CT) screening for lung cancer in high‐risk individuals has increased the detection rate of small PLLs.^6^, ^7^ However, most PLLs on low‐dose CT screening are found to be benign, which makes histological diagnosis with accurate and safe tissue sampling methods, such as RP‐EBUS‐TBB, even more important. In particular, there may occasionally be mediastinal LN enlargement even with small PLLs on a CT scan. Contrast‐enhanced CT scans and positron emission tomography (PET) remain unsatisfactory for evaluating mediastinal LNs;^1^ consequently, some patients require histological examinations for both PLLs and mediastinal LNs. To date, little is known about the combined procedure using RP‐ and CP‐EBUS. Therefore, we aimed to investigate the efficacy and safety of concurrent bronchoscopy using RP‐ and CP‐EBUS.

## METHODS

Between January 2020 and March 2021, the bronchoscopy database at Pusan National University Hospital, Republic of Korea, was used to identify the clinical outcomes of the combined procedure with RP‐ and CP‐EBUS. During the study period, 617 patients received RP‐EBUS‐TBB to evaluate PLLs. Two of these patients were excluded because PET was performed at another hospital, and could not be analyzed by nuclear radiologists. Another 12 patients were excluded because RP‐EBUS‐TBB was performed for a rebiopsy to detect genetic mutations in the lung cancer.^8^ As a result, 603 patients were included this study. Patients who underwent RP‐EBUS‐TBB only were categorized as the RP‐EBUS group, and those who underwent a combined procedure with RP‐EBUS‐TBB and CP‐EBUS‐TBNA simultaneously were categorized as the combination group. For subgroup analyses, based on the results of the bronchoscopic biopsy, the patients diagnosed with lung cancer in the combination group were classified into three subgroups: group A, diagnosed with lung cancer on both RP‐EBUS‐TBB and CP‐EBUS‐TBNA; group B, diagnosed with lung cancer only on RP‐EBUS‐TBB; and group C, diagnosed with lung cancer only on CP‐EBUS‐TBNA. This retrospective study was approved by the institutional review board (IRB) of Pusan National University Hospital (IRB no. 2102–004‐099). All data were fully anonymized and the ethics committee waived the requirement for informed consent due to the retrospective nature of the study.

### CT and PET analysis

PLLs were defined as any lung lesion existing beyond the segmental bronchus in the axial plane of the thin‐section CT scan.^9^ The mean PLL diameter was calculated as the mean of the longest and perpendicular diameters, measured on axial CT scans. The distance from the pleura to PLL was measured as the shortest distance between the visceral pleura and PLL. Positive bronchus sign was defined as the presence of peripheral bronchi, leading directly to the PLL on a thin‐section CT scan, as assessed by pulmonary physicians.^10^ PLLs were classified as solid, mixed, and ground‐glass opacities based on CT attenuation.^11^ A chest radiologist reviewed the chest CT images and determined the LN status; a short‐axis diameter ˃10 mm was defined as abnormal, and classified as metastasis.^12^, ^13^ PET was assessed by an experienced nuclear physician, and LN metastasis was determined visually (i.e., LNs with abnormal fluorodeoxyglucose [FDG] uptake [greater than the mediastinal blood pool uptake], regardless of size, were considered metastatic).^14^

### Bronchoscopic procedures

All procedures were performed under conscious sedation with intravenous midazolam and fentanyl. RP‐EBUS‐TBB was performed according to Kurimoto's standard technique, with or without virtual bronchoscopy navigation (Lung point; Broncus Medical Inc.).^4^, ^15^, ^16^ The bronchoscope was introduced as close to the PLL as possible using a 4.0 mm flexible bronchoscope (BF‐P260F; Olympus). Then, a 20 MHz radial miniature probe (UM‐S20‐17S; Olympus), covered with a guide sheath (K‐201; Olympus), was introduced via the working channel of the bronchoscope, and the sonographic findings of the PLL were classified as within, adjacent to, or outside the lesion.^17^, ^18^ When the PLL was found precisely using a radial miniature probe, forceps biopsy and brush cytology were performed under fluoroscopic guidance.

**FIGURE 1:**
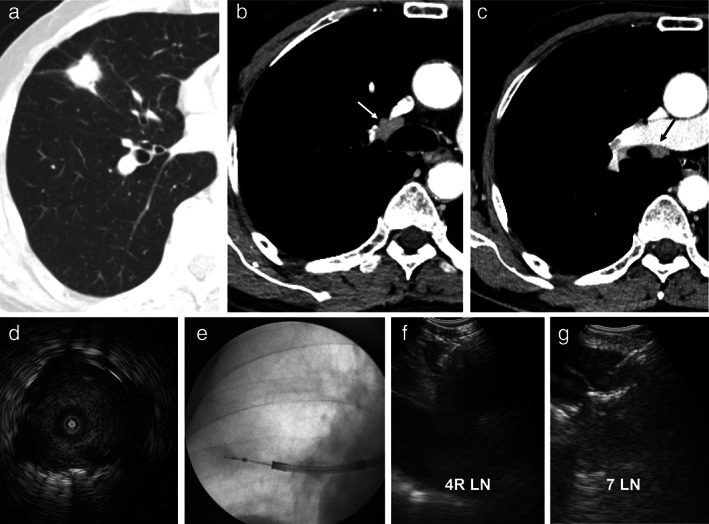
A representative case of the combined procedure with RP‐ and CP‐EBUS. (a) An 18‐mm peripheral lung lesion was found in the right middle lobe. (b, c) Axial CT images with a mediastinal window showing enlargements in 4R (white arrow) and 7 (black arrow) lymph nodes. (d) The peripheral lung lesion was found to be “within” on the ultrasonographic image during RP‐EBUS. (e) Transbronchial biopsy was performed under fluoroscopy guidance. (f, g) Subsequent transbronchial needle aspiration with CP‐EBUS was performed at 4R and 7 lymph nodes. As a result, an adenocarcinoma was found in the right middle lobe, but there was no evidence of mediastinal lymph node metastasis. The patient underwent thoracoscopic surgery and was diagnosed with stage IA lung adenocarcinoma (T1bN0M0). CP‐EBUS, convex probe endobronchial ultrasound aspiration; CT, computed tomography; LN, lymph node; RP‐EBUS, radial probe endobronchial ultrasound.

When mediastinal LN sampling using CP‐EBUS was required, based on the CT scan or PET, it was followed by a RP‐EBUS‐TBB procedure. Using an ultrasound bronchoscope (BF‐UC260FW; Olympus), CP‐EBUS‐TBNA was performed at the mediastinal LNs with a clinical suspicion of metastasis. A representative case of the combined procedure with RP‐ and CP‐EBUS is shown in Figure 1.

### Diagnosis

When bronchoscopy procedures using RP‐EBUS, with or without CP‐EBUS, failed to confirm the diagnosis, an additional surgical biopsy, percutaneous needle aspiration, or repeat bronchoscopy was performed after obtaining consent. Benign lesions, not otherwise specified, were defined as inflammatory cells only or a benign pathology, such as anthracotic pigmentation, on the biopsy specimen, with no changes on CT over at least 12 months of follow‐up.

### Statistical analysis

All results are reported as numbers (percentages) for categorical variables and as the mean ± standard deviation for continuous variables. Data were compared using the chi‐squared or Fisher's exact test for categorical variables, and independent *t*‐test or Wilcoxon rank‐sum test for continuous variables. The diagnostic yield of the EBUS procedure was calculated by dividing the number of successful diagnoses by the total number of procedures. SPSS Statistics for Windows version 22.0 (IBM Corp.) was used for statistical analyses.

## RESULTS

### Study population

The RP‐EBUS and combination groups consisted of 506 (83.9%) and 97 (16.1%) participants, respectively. The baseline characteristics of the two study groups are shown in Table 1. The proportion of the male sex and the mean age in the RP‐EBUS group were significantly lower compared to the combination group (61.3% vs. 82.5% males, *p* < 0.001; mean age: 68.1 vs. 70.4 years, *p* = 0.021). Compared to the RP‐EBUS group, the combination group had larger mean PLL diameters (29.7 vs. 36.9 mm, *p* < 0.001), a greater proportion had a positive bronchus sign (84.2% vs. 93.8%, *p* = 0.022), and a greater proportion had solid PLLs (86.2% vs. 94.8%, *p* = 0.039) on thin‐section CT.

**TABLE 1:** Baseline characteristics

Variables	RP‐EBUS group (*n* = 506)	Combination group^a^ (*n* = 97)	*p*‐value
Male sex	310 (61.3)	80 (82.5)	<0.001
Age, years	68.1 ± 10.1	70.4 ± 8.9	0.021
Location of PLL			0.251
Right upper lobe	154 (30.4)	24 (24.7)	
Right middle lobe	34 (6.7)	11 (11.3)	
Right lower lobe	108 (21.3)	26 (26.8)	
Left upper lobe	138 (27.3)	21 (21.6)	
Left lower lobe	72 (14.2)	15 (15.5)	
Mean diameter of PLL, mm	29.7 ± 14.2	36.9 ± 17.2	<0.001
Distance from pleura to PLL, mm	12.9 ± 14.4	12.8 ± 14.9	0.922
Positive bronchus sign on CT scan	426 (84.2)	91 (93.8)	0.022
Character of PLL on CT scan			0.039
Solid	436 (86.2)	92 (94.8)	
Mixed	68 (13.4)	5 (5.2)	
Ground‐glass opacity	7 (1.4)	0 (0)	

Abbreviations: CT, computed tomography; PLL, peripheral lung lesion; RP‐EBUS, radial probe endobronchial ultrasound.

^a^ The combination group included patients who underwent a combined procedure using RP‐EBUS and convex probe EBUS.

### Diagnostic yield

There was significant difference in the diagnostic yield of RP‐EBUS‐TBB between RP‐EBUS and combination groups (70.0% vs. 81.4%, *p* = 0.021). The overall diagnostic yields of bronchoscopic procedure in the RP‐EBUS and combination groups were 70.0 and 90.7%, respectively (*p* < 0.001) (Table 2). In addition to RP‐EBUS‐TBB, CP‐EBUS‐TBNA increased the diagnostic yield in the combination group by 9.3%. Table 3 shows the final clinical diagnoses of the two study groups.

**TABLE 2:** Diagnostic yields and safety profiles

	RP‐EBUS group (*n* = 506)	Combination group^a^ (*n* = 97)	*p*‐value
Diagnostic yields			
RP‐EBUS‐TBB	70.0%	81.4%	0.021
Overall diagnostic yield	70.0%	90.7%	<0.001
Complications			
Pneumothorax	5 (1.0)	1 (1.0)	0.310
Infection	2 (0.4)	0 (0.0)	0.651
Massive bleeding	0 (0.0)	0 (0.0)	N/A
Life threatening event	0 (0.0)	0 (0.0)	N/A
Overall complications	7 (1.4)	1 (1.0)	0.766

Abbreviations: CP‐EBUS‐TBNA, transbronchial needle aspiration using convex probe EBUS; RP‐EBUS, radial probe endobronchial ultrasound; RP‐EBUS‐TBB, transbronchial biopsy using RP‐EBUS.

^a^ The combination group included patients who underwent a combined procedure using RP‐EBUS and convex probe EBUS.

**TABLE 3:** Clinical diagnoses

Variables	RP‐EBUS group (*n* = 506)	Combination group^a^ (*n* = 97)
Malignant disease		
Lung cancer		
Adenocarcinoma	230 (45.5)	48 (49.5)
Squamous cell carcinoma	69 (13.6)	28 (28.9)
Non‐small cell lung cancer, NOS	22 (4.4)	4 (4.1)
Small cell lung cancer	19 (3.8)	13 (13.4)
Metastasis from extrathoracic malignancy		
Renal cell cancer	2 (0.4)	0 (0.0)
Biliary cancer	1 (0.2)	0 (0.0)
Lymphoma	0 (0.0)	1 (1.0)
Benign disease		
Pulmonary tuberculosis	17 (3.4)	0 (0.0)
Aspergilloma	6 (1.2)	0 (0.0)
Necrotizing pneumonia	3 (0.6)	0 (0.0)
Cryptococcosis	2 (0.4)	0 (0.0)
Benign disease, NOS^b^	12 (2.4)	3 (3.1)
Undiagnosed	123 (24.3)	0 (0.0)

Abbreviations: NOS, not otherwise specified; RP‐EBUS, radial probe endobronchial ultrasound.

^a^ The combination group included patients who underwent a combined procedure using RP‐EBUS and convex probe EBUS.

^b^ Benign disease, NOS was defined when inflammatory cells were detected only or a benign pathology was evident in biopsy specimens, with no change over at least 12 months of follow‐up.

### Safety profile

There was a significant difference in the mean procedure time between the RP‐EBUS and combination groups (19.6 vs. 29.8 min, *p* < 0.001). The mean doses of midazolam and fentanyl, administered for conscious sedation, were significantly lower in the RP‐EBUS than the combination group (3.1 vs. 3.5 mg, *p* < 0.001, and 45.8 vs. 58.5 μg, *p* < 0.001 for midazolam and fentanyl, respectively). However, there were no differences in the overall complication rates between the RP‐EBUS and combination groups (1.4 and 1.0%, respectively; *p* = 0.766) (Table 2). The most frequent complication was pneumothorax, which occurred in five patients in the RP‐EBUS group and one patient in the combination group.

### Clinical outcomes of the combination group

**FIGURE 2:**
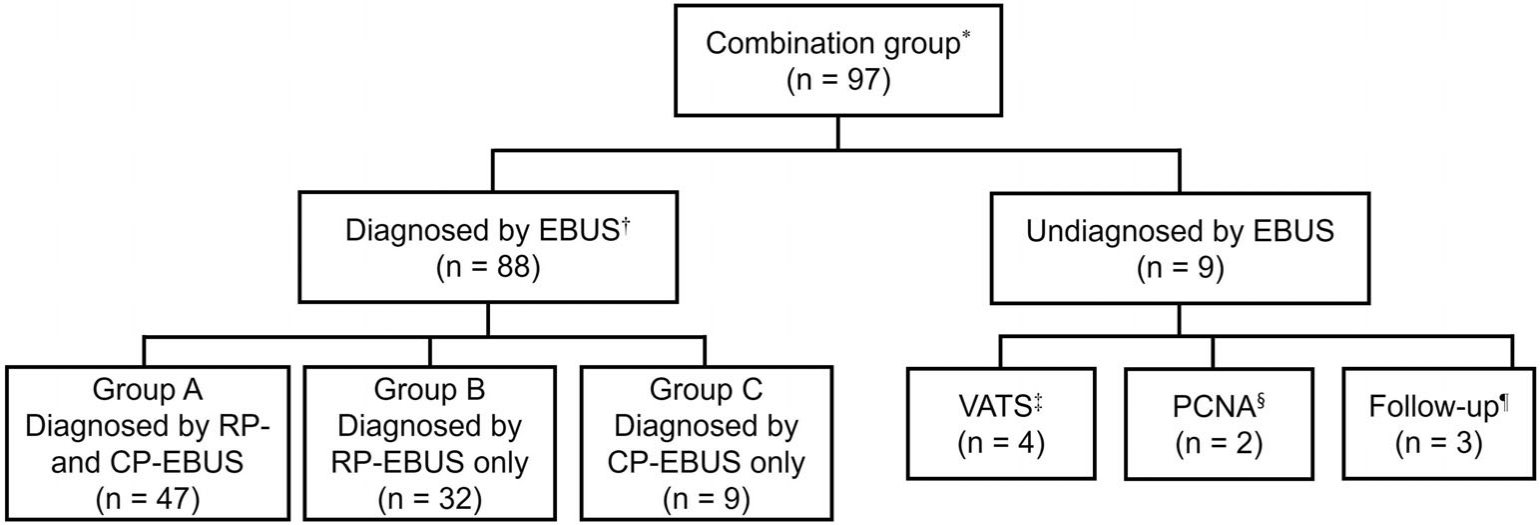
Diagnostic modalities of the combination. *The combination group included patients who underwent a combined procedure using RP‐ and CP‐EBUS. †All 88 patients diagnosed using EBUS were diagnosed with lung cancer. ‡Among the patients diagnosed using VATS, three were diagnosed with lung cancer, while one was diagnosed with lymphoma. §Both patients diagnosed using PCNA were diagnosed with lung cancer. ¶All three patients followed‐up had no changes in the lesion over a minimum of 12 months. CP‐EBUS, convex probe endobronchial ultrasound; PCNA, percutaneous needle aspiration; RP‐EBUS, radial probe endobronchial ultrasound; VATS, video‐assisted thoracic surgery.

In the combination group, subgroups A, B, and C consisted of 47 (48.5%), 32 (33.0%), and nine (9.3%) participants, respectively (Figure 2). Table 4 shows the clinical outcomes of the subgroups. In group A (47 patients histologically diagnosed from both the primary tumor and mediastinal LNs), non‐small cell lung cancer (NSCLC) and small cell lung cancer (SCLC) were found in 39 and eight patients, respectively. Among the 39 NSCLC patients in group A, the number of aspirated N1, N2, and N3 LNs were three, 47, and five, respectively; clinical stages III and IV were diagnosed in 37 and two patients, respectively. Seventeen NSCLC patients underwent thoracoscopic surgery, with or without neoadjuvant chemotherapy, and there were no false positive or negative results in the 28 LNs examined using CP‐EBUS. In addition, all eight SCLC patients in group A were found to have an extensive stage.

**TABLE 4:** Clinical outcomes of groups A, B, and C

Diagnosis	Group A (*n* = 47)	Group B (*n* = 32)	Group C (*n* = 9)
Non‐small cell lung cancer	39/47	29/32	7/9
Clinical stage			
I	0/39 (0)	8/29 (27.6)	0/9 (0)
II	0/39 (0)	19/29 (65.5)	0/9 (0)
III	37/39 (94.9)	0/29 (0)	7/9 (100.0)
IV	2/39 (5.1)	2/29 (6.9)	0/9 (0)
Treatment			
Stereotactic body radiation therapy	0/39 (0)	2/29 (6.9)	0/9 (0)
Thoracoscopic surgery	17/39 (43.6)	19/29 (65.5)	5/9 (71.4)
Concurrent chemoradiation therapy	9/39 (23.1)	0/29 (0)	1/9 (14.3)
Palliative chemotherapy	8/39 (20.5)	2/29 (6.9)	1/9 (14.3)
Follow‐up loss	5/39 (12.8)	6/29 (20.7)	0/9 (0)
Small‐cell lung cancer	8/47	3/32	2/9
Limited stage	0/8 (0)	2/3 (66.7)	0/2 (0)
Extensive stage	8/8 (100)	1/3 (33.3)	2/2 (100)

Among the 29 NSCLC patients in group B, the number of aspirated N1, N2, and N3 LNs were 12, 28, and five, respectively, and all LNs were negative for malignancy in CP‐EBUS‐TBNA specimens. Consequently, clinical stages I, II, and IV were diagnosed in eight, 19, and two patients (due to unexpected brain metastasis), respectively. In total, 19 patients underwent thoracoscopic surgery with LN dissection, and four of the 32 LNs, aspirated using CP‐EBUS, were found to be false‐negatives. Unexpected N2 LN metastasis was found in the surgical specimen in one patient. In group B, three patients were diagnosed with SCLC, two with limited stage SCLC and one with extensive stage SCLC.

In group C, nine patients were diagnosed using CP‐EBUS‐TBNA only, and all RP‐EBUS‐TBB results were found to be false‐negative. Among the seven NSCLC patients in group C, the number of aspirated N1, N2, and N3 LNs were two, eight, and one, respectively. All patients were eventually diagnosed with clinical stage III NSCLC. Thoracoscopic surgery was performed in five patients, and there were no false‐negative or ‐positive results in the eight LNs examined using CP‐EBUS.

In the combination group, 41 NSCLC patients (54.7%) underwent surgery. Using the surgical specimen as the reference, the sensitivity, specificity, positive predictive value, negative predictive value, and accuracy of CP‐EBUS‐TBNA were determined to be 86.2, 100, 100, 87.5, and 93.0%, respectively.

## DISCUSSION

In the present study, we demonstrated that a combined procedure with RP‐ and CP‐EBUS was feasible for both histological diagnoses of primary tumors and mediastinal staging for suspected lung cancer. Our results demonstrate that CP‐EBUS‐TBNA, performed after RP‐EBUS‐TBB, improves the diagnostic yield of bronchoscopy. In addition, there was no difference in complication rates between the RP‐EBUS and combination groups (1.4% vs. 1.0%). To the best of our knowledge, this is the first study to investigate the clinical use and safety of the combined procedure with RP‐ and CP‐EBUS.

Adenocarcinoma is known for early spread to adjacent LNs; the risk of occult LN metastases is reported to be more than twice as high as other histologies.^19^ Moon et al.^20^ reported N1 or N2 LN metastases in 15% of 276 peripheral adenocarcinoma patients (median size: 2.3 cm). Luo et al.^21^ reported that patients with tumor sizes <1 cm or pure ground‐glass lesions were unlikely to have metastatic LNs (1.9% and 0%, respectively). In their analyses, although the frequency was lower than NSCLCs located in the medial half, 8% of the NSCLCs in the lateral half were also accompanied by LN metastases. The incidence of peripheral adenocarcinomas, which are the most common indication for RP‐EBUS‐TBB, has recently surpassed that of squamous cell carcinoma worldwide.^22^ Therefore, accurate evaluation of the mediastinal LNs, as well as the primary tumor, is essential for treatment planning, particularly for a combined procedure with RP‐ and CP‐EBUS.

In addition to mediastinal staging, our results show that CP‐EBUS‐TBNA, performed after RP‐EBUS‐TBB, increased the diagnostic yield of bronchoscopy. Previous studies reported that the diagnostic yield of RP‐EBUS‐TBB was 70%–80%,^3^, ^23^ which was similar to the yield in the combination group in this study. In the present study, additional CP‐EBUS‐TBNA for mediastinal staging increased the diagnostic yield by 9.3% (false‐negative results in RP‐EBUS‐TBB). This suggests that a combined procedure with RP‐ and CP‐EBUS complemented the diagnostic yield and improved the accuracy of mediastinal staging.

Previous studies have reported that the sensitivity and specificity of CP‐EBUS‐TBNA were 89–99% and 100%, respectively.^24^, ^25^, ^26^, ^27^ The addition of CP‐EBUS‐TBNA to RP‐EBUS‐TBLB may increase physician fatigue and decrease patient cooperation due to prolonged sedation, which may decrease the overall accuracy of mediastinal staging. However, our findings suggest that the diagnostic yield of the subsequent CP‐EBUS‐TBNA was similar to that in previous studies about the performance of CP‐EBUS‐TBNA in mediastinal staging.

Çağlayan et al.^28^ and Asano et al.^28^, ^29^ reported 0.2%–0.5% procedure‐related complication rates for CP‐EBUS‐TBNA. Hayama et al.^30^ reported that the overall complication rate for RP‐EBUS‐TBB was 1.3% in an analyses of 933 PPLs. Although both CP‐EBUS‐TBNA and RP‐EBUS‐TBB are known to be safe procedures, there could be concerns about the safety of their combination. However, our results demonstrated no significant difference in procedure‐related complications between the RP‐EBUS and combination groups (1.4% vs. 1.0%).

This study has some limitations. First, it was a retrospective study conducted at a single institute. Although the bronchoscopy database collected information for consecutive patients, potential selection bias could have influenced our results. In particular, there were significant differences in the baseline characteristics of the two groups. Second, RP‐EBUS‐TBB was performed conventionally, without the assistance of advanced bronchoscopy modalities, such as robotic bronchoscopy and cone‐beam CT, which could increase the diagnostic yield of the procedure. Third, the number of patients in the combination group, subgroup C was relatively small; thus, the findings may not be generalizable. Multicenter prospective studies with a larger number of participants are required to verify our findings.

In conclusion, the combined procedure with RP‐ and CP‐EBUS is feasible and safe. In addition to mediastinal staging, additional CP‐EBUS‐TBNA increased the overall diagnostic yield of bronchoscopic procedures by 9.3%.

## CONFLICT OF INTEREST

All authors declare that they have no conflicts of interest relevant to this study.
